# Migrations in Italy and Perceptions of Ethnic Threat

**DOI:** 10.1007/s12134-022-00985-8

**Published:** 2022-09-16

**Authors:** Annamaria Nese

**Affiliations:** Department of Economics and Statistics, CELPE, University of Salerno, Via Giovanni Paolo II, 132 - 84084 Fisciano (SA), Italy

**Keywords:** Immigration, Anti-immigrant sentiment, Ethnic threat, Competition theory, D04, J15, J61

## Abstract

This work investigates anti-immigrant sentiment in Italy and to what extent any “perceived ethnic threat” is influenced by the actual presence of immigrants. Whereas previous studies in the Italian context provide evidence for various social and psychological explanations of anti-immigrant sentiment, this work underlines the role of economic factors focusing on competition theory as main theoretical explanation. The analysis examines microdata obtained from the European Social Survey and from the Labour Force Survey conducted in 2016. In line with the economic perspective, the results suggest that the percentage of unemployed immigrants—rather than just the number of immigrants—significantly increases natives’ perceptions of an “ethnic threat.”

## Introduction

Many developed countries have seen a rise in anti-immigrant sentiment, in part due to worsening economic conditions and an increasing number of immigrants and refugees. A significant amount of research has analyzed the consequences of this phenomenon since understanding natives’ concerns about and sentiment towards immigrants is crucial in formulating adequate immigrant integration policies (Fetzer, 2000, 2011; Finseraas, 2012; Manevska & Achterberg, 2011; Olzak, 1992; Papademetriou & Banulescu-Bogdan, 2016).

Overall, the consequences of immigration can differ significantly both across and within countries as a result of different historical and local socioeconomic contexts. Some countries, for example, already have sizeable ethnic minorities because of postcolonial migration flows (e.g., France and the UK) or active policies of labor recruitment (e.g., in Germany). Other countries are characterized by the presence of autochthonous minorities (e.g., in eastern Europe) while others have only recently become the destinations of growing numbers of migrants (e.g., Italy and Spain).

Based on Gallup’s World Poll (2012–2014), Esipova et al. (2015) outline a sharp divergence in opinions among residents in northern and southern Europe. The majority of adults in northern Europe (e.g., in Germany)—except for those in the UK—would like immigration levels to either stay the same or increase, while most residents in southern Europe would prefer to have lower levels of immigration in their countries. Public sentiment is even more negative in Mediterranean countries that are the entry points to the continent for many migrants and refugees (Greece, Malta, Italy). Papademetriou and Banulescu-Bogdan (2016) suggest^1^ that the degree of immigration itself or economic downturns do not necessarily determine anti-immigrant sentiment; rather, sudden flows of immigrants can increase public concerns in countries less accustomed to migration, particularly when segments of the local population are facing economic hardship. Hence, not only the characteristics of immigration (size and composition) matter, but also specific economic, social, and political conditions in the host society.

This study focuses on public sentiment towards immigrants in Italy, considering only one country—as opposed to a large number of countries—could advance our understanding of the factors shaping public opinion by reducing the heterogeneity in political and socioeconomic factors.

Italy has only recently become a country of immigration: in the first decade of the 2000s, the share of foreigners in the population more than tripled, growing from 2.3% in 2002 to 6.84% in 2011.^2^ High values were registered in northern Italy (with particularly high values in Piedmont, Lombardy, and Veneto) and central Italy (with peaks in Emilia Romagna, Tuscany, and Latium), while in southern Italy, the share of immigrants remained much lower, rising from 0.8 to 2.7%. In conjunction with the so-called Arab Springs, the number of asylum seekers and refugees went from 16,844 in 2012 to 188,084 in 2016 (Colucci, 2018), while in 2016, the percentage of foreigners in the population was around 8%.

Immigrants to Italy mainly come from non-European Union and low-income countries. According to data provided by the Italian National Institute of Statistics (Istat), in 2016, the top 10 countries of origin were (in descending order) Romania, Albania, Morocco, China, Ukraine, the Philippines, India, Moldova, and Bangladesh.

Political and media attention has mainly focused on the issues of illegal immigration, on security and crime problems, on the management of the refugee crisis, less on the ordinary immigration management policy, and on the integration of foreign citizens^3^ (Pugliesi, 1995; Colombo & Sciortino, 2004; Campani, 2008; Colucci, 2018). Italy’s approach to integration in the years 2014–2019 has been classified as “Halfway favorable” on the basis of the by the Migrant Integration Policy Indicators (MIPEX); however, similar ratings have been reported in France, the Netherlands, Germany, and the UK.^4^

Overall, concerns about the increasing immigration flows and the global refugee issue in the last decades, in addition to the massive unemployment rate and economic crisis, have characterized the social and political debate. In addition, the influx of religiously different newcomers could be a further source of anxiety in a country that represents the center of Catholicism.

Previous studies on Italy underline the effect of interpersonal contact on anti-immigrant sentiment (Panichella & Abrosini, 2018; Salvati et al., 2019). This study, instead, aims to explain public opinion on immigration with particular reference to the economic factors outlined in labor market competition theory. Based on previous theoretical and empirical studies, this work hypothesizes that attitudes towards immigrants are determined by the individual characteristics of natives as well as by contextual factors. Hence, it refers to two levels of analysis: micro and macro. The micro level focuses on natives’ socioeconomic conditions arguing that lower-class people with a weaker position in the labor market are more likely to perceive immigrants as a threat and to manifest anti-immigrant sentiments. The macro level focuses on regional characteristics such as the numbers of immigrants, the level of unemployment, and local per capita income.

This work also investigates endogeneity issues regarding the migration flows across Italy’s regions. The empirical approach adopted relies on a well-known instrument proposed by Card (2001), and slightly modified by Cortes and Pan (2015), which exploits the fact that immigrants generally move to geographical areas in which their compatriots have already settled. This instrument was proposed by Barone et al. (2016) and Caselli et al. (2020) in order to investigate the role of immigration in shaping voting behavior in Italy.

The empirical analysis relies on microdata drawn from the eighth wave of the European Social Survey, conducted in 2016 (ESS2016), since the data for Italy are aggregated at the NUTS2/region level.

The paper is organized as follows. Section 2 reviews theoretical explanations for anti-immigrant sentiment and introduces the paper’s research questions. Section 3 describes the data and the estimation methods. Section 4 presents and discusses the results and Sect. 5 draws some conclusions.

## Theoretical Background

Theories on the formation of attitudes towards immigrants include two important strands of research: social-psychological explanations and economic self-interest-based explanations. This work places itself in the second strand of research (see Sect. 2.2). Section 2.1, however, contains a short review of the various theoretical approaches, with particular reference to the role of “threat” in determining attitudes towards immigrants.

### The Role of Threat

Attitudes towards immigrants can be analyzed with reference to a wide theoretical perspective concerning the role of threat in determining prejudices. Sociologists and psychologists have highlighted that migration-related attitudes are largely determined by perceptions of threat and competition, heightened uncertainty, feelings of a lack of control, and a rise in authoritarianism.

Based on a literature review, Stephan et al. (1999) suggest that negative attitudes are mainly determined by four basic kinds of threats: *Realistic threat*, *Cultural threat*, *Intergroup anxiety*, and *Intergroup stereotypes*. Realistic threat is a threat to the existence or well-being of the in-group due to competition for scarce resources. Symbolic threats concern differences among groups in terms of morals, values, norms, standards, beliefs, and attitudes. Anxiety is likely to occur when people enter a previously homogeneous group and are concerned about negative outcomes for themselves, such as being embarrassed, rejected, ridiculed, or exploited (Stephan and Stephan, 1985). Negative stereotypes attribute negative traits to explain the behavior of out-group members, thus justifying discrimination against them (Eagly & Mladinic, 1989; Esses et al., 1993).

Empirical research on whether and how threat perceptions and emotions affect attitudes towards immigrants and refugees is increasingly common (Esses et al., 2010; Jetten & Esses, 2018), mainly relying on laboratory experiments. The generalized feeling of threat has been found as a determinant of xenophobia (Clissold et al., 2020) and prejudice towards immigrants (Murray & Marx, 2013). Feelings of a lack of control have also been found to be correlated with negative attitudes towards immigrants, either on their own (Harell et al., 2017) or as a moderator of threat perceptions (Greenaway et al., 2014). In lab experiments in Australia and Israele, Canetti et al. (2016) showed that threat perceptions of out-group members shape conservative political ideologies and support for exclusionary policies.

Extending the threat theory, Tartakovsky and Walsh (2015) suggested that a local population’s perception of immigrants as threatening or beneficial depends on *personal value preferences*; for example, people who value social security and tradition highly tend to perceive immigrants as threatening to the host country.

Rustenbach (2010) adds to the empirical literature by highlighting that individuals with high levels of *interpersonal trust* fear the arrival of immigrants less.

While lines of research focus on the circumstances under which threat and fear might determine ethnic conflicts, others highlight factors that could help to reduce fears and prejudices. *Contact theory* (Allport, 1954), for example, suggests that more frequent contact between natives and ethnic minorities reduces racial and ethnic prejudices. This argumentation is confirmed by many authors (Andreescu, 2017; Pettigrew & Tropp, 2006, 2008; Voci & Hewstome, 2003); however, other results indicate a positive correlation (or no significant correlation) between the proportion of immigrants and anti-immigrant sentiment (Markaki & Longhi, 2013; Rustenbach, 2010; Swart et al., 2011; Turner et al., 2007; Visintin et al., 2017). The main difficulty associated with such empirical analyses is that the available data—usually the percentage of immigrants in a country—may not adequately represent the frequency of interpersonal contact between ethnic minorities and natives.

In addition to contact theory, *cultural marginal theory* highlights the impact of non-economic factors in explaining public opinion towards immigration. According to this theory, people with cultural and ethnic ties to immigrants—as well as individuals who have suffered discrimination due to race, gender, economic conditions, etc.—are less likely to express anti-immigrant sentiments (Fetzer, 2000, 2011). Empirical tests consistent with this theory are reported in numerous works (Andreescu, 2011, 2017; Markaki & Longhi, 2013).

### Self-Interest and Ethnic Competition Theory; Previous Empirical Evidence

The economic literature on anti-immigrant sentiment based on self-interest has mainly rooted in the *ethnic competition theory* (Olzac, Olzak, 1992; Fetzer, 2000; Manevska & Achterberg, 2011). The competition theory of ethnic/racial conflict emphasizes that as ethnic and racial groups enter a population, competition for limited resources is likely to increase. The core idea is that “competition for resources leads to attempts at exclusion of one group by another” (Olzak, 1992, p. 163). Hence, lower-class people with a weaker position in the labor market are more likely to report negative views of minorities since they see themselves in a stronger competition with immigrants not only for low-skill jobs but also for affordable housing and social services. At a macro level, increasing discriminatory behaviors are likely in regions characterized by (i) poor economic conditions or (ii) a high percentage of immigrants threatening to take residents’ jobs and lower their wages.

Adopting a micro-level approach, most empirical results confirm that low-educated, working-class, or unemployed people are more likely to express anti-immigrant sentiment (Andreescu, 2011; Card et al., 2005; Manevska & Achterberg, 2011; Markaki & Longhi, 2013; Rustenbach, 2010). Empirical tests, however, are not always consistent with these assertions (Fetzer, 2000; Sobczak, 2007; Jolly & Di Giusto, 2014). Competition in the labor market is not the only explanation based on self-interest. Dustmann and Preston (2007), for example, argue that anti-immigrant sentiment is higher among those who believe that immigration is responsible for placing additional burdens on the welfare system, and Barone et al. (2018) underline the likely relation to overloaded public services.

When adopting a macro-level approach, once again, empirical findings are often weak and inconclusive (Card et al, 2005; Jetten et al., 2015; Moscatelli et al., 2014; Papademetriou & Banulescu-Bogdan, 2016). One likely explanation of such results is related to an endogeneity issue in that wealthier regions are also more likely to experience immigration. Furthermore, the association between discriminatory behaviors and the size of the immigrant population may not provide an adequate test for competition theory; Schneider (2008), for example, relies on the actual percentage of immigrants among the poor in a country; Markaki and Longhi (2013) report the percentage of unemployed immigrants. Most works, however, are simply based on the percentage of immigrants from non-western countries (Manevska & Achterberg, 2011).

### Relation to Literature and Research Hypotheses

Relatively few studies have examined anti-immigrant sentiment in Italy based on survey data.^5^ Panichella and Ambrosini (2018) and Salvati et al. (2019) investigated whether and to what extent social contact with immigrants influences discriminatory attitudes towards them. Overall, the authors’ results are consistent with contact theory and confirm the importance of education in reducing prejudice and intolerance. Russo et al. (2019) focused on the relationship between immigrants’ group size and ethnic prejudice and did not report any significant evidence consistent with competition theory, although they highlighted the correlation between some political ideologies and individual attitudes towards immigrants.^6^

The specific aim of this research is to understand people’s perception of the ethnic threat in Italy, with particular reference to the economic aspects underlined by competition theory. Taking into account theoretical and empirical literature, this work identifies testable hypotheses on what characteristics of the regional contexts (at macro-level) and of individuals (at micro-level) are likely to affect anti-immigrant attitudes.

The main research hypotheses are the following:

- Hypothesis 1. The share of unemployed immigrants at regional level (rather than the mere number of immigrants) is expected to increase hostility towards ethnic minorities.
- Hypothesis 2. Respondents’ education and socioeconomic status are expected to influence their opinion about immigration. Consistently with our argumentation, a better personal economic and labor market situation would promote more positive attitudes towards immigrants.
- Hypothesis 3. The characteristics of a region in terms of per capita product and unemployment rate are expected to be correlated with anti-immigrant sentiments since worse economic conditions increase competition among social groups for limited resources.

As we will see in more detail in Sects. 3.2 and 4.1, other individual predictors—such as age, gender, personal value preferences, and beliefs—will be included as “control variables” in the empirical analysis since their exclusion could affect our main results; since these variables are of interest, the results will be reported and discussed according to the literature.

## Methods, Variables, and Data

### Methods

The analysis was performed on a representative sample drawn from the eighth wave of the European Social Survey conducted in 2016 (ESS2016). The sample includes individuals aged eighteen and over (*N* = 2325) living in Italy, regardless of nationality or citizenship; 207 respondents were foreign born or defined themselves as ethnic minority members.^7^

The research focuses on both natives’ and foreigners’ perceptions of ethnic threat since the effects of immigration are typically felt locally by all residents (for example, in terms of the labor market and healthcare resources). However, given that individuals with an immigrant background may have different opinions about immigration, this work provides additional estimates excluding ethnic minorities and non-natives from the sample.

Anti-immigrant sentiment was measured based on answers to three questions included in the ESS2016. Specifically, respondents shared their views on the following sentences: (i) “Would you say it is generally bad or good for country’s economy that people come to live here from other countries?”; (ii) “Would you say that country’s cultural life is generally undermined or enriched by people coming to live here from other countries?”; and (iii) “Is country made a worse or a better place to live by people coming to live here from other countries?” In the last case, the fear of an “ethnic threat” should include economic and cultural concerns about immigration as well as other issues (e.g., increased criminality and increased competition for public services). The answers were coded on a scale of 0 to 10 (lower values indicate lower propensity to accept immigration).

This empirical analysis relies on ordinary least squares (OLS) estimates where the dependent variable is obtained by averaging the scores reported for the three survey questions, which facilitates interpretation of the effect sizes of each regressor.

To check robustness, a parallel analysis was performed using each of the three questions reported above. Since the answers are coded on a scale of 0 to 10, the estimates were based on ordered probit models. The results of these additional analyses, which are available on request, confirm the main results presented in Sect. 4.2 below.

Empirical results on the impact of the number of immigrants on anti-immigrant sentiments have often been found to be weak and inconclusive. This could be due to endogeneity in the number of immigrants.

Endogeneity could be determined by omitted-variable problems, reverse causality, or measurement errors. For example, unobserved local productivity shocks may lead to both an increase in the number of immigrants and lower competition in the labor market, which—according to competition theory—would entail lower perceptions of ethnic threat among residents. An obvious solution may be to include regressor controls for local economic conditions, such as the gross domestic product or unemployment rate. Reverse causality could also arise because immigrants may choose to live in regions where residents are less averse to foreigners. Finally, measurement errors are likely to affect the regressor since the data on immigrants rarely account for irregular immigration.

The method of instrumental variable (IV) and two-stage least squares estimates (2SLS)^8^ provide a solution to address potential endogeneity in the number of immigrants; more specifically, the ordinary least squares (OLS) estimates can be compared to 2SLS in order to test for endogeneity. To build an instrumental variable, it is necessary to identify an “exogenous source of variation” in the local share of immigrants; this variation is not necessarily random but must be uncorrelated with the unobserved component of attitudes towards immigrants, which is conditional on the regressors.

The IV proposed in this paper is based on the distribution across Italy’s regions (NUTS2) “in the past” of immigrants according to their origins; it should provide the required source of variation in the “current” share of immigrants at regional level. This instrumental variable is drawn from previous studies on the effects of migrations, such as Card (2001), Cortes and Pan (2015), Barone et al. (2016), Bratti and Conti (2017), and Caselli et al. (2020), and relies on the tendency of immigrants to migrate to areas where communities of immigrants from the same country of origin have already settled. In fact, immigrant networks usually provide information on the migration process itself, hospitality, help, and information on local jobs and social services.

Formally, the instrumental variable (IV) is computed for each region “s” as follows:

$${IV}_{S}={\sum }_{c=1}^{N}\frac{{immigrants}_{cs,2005}}{{immigrants}_{c,2005}}*{immigrants}_{c2005-s}$$

where *s* denotes the Italian region where each interviewed individual lives, and *c* denotes immigrants’ country of origin.^9^ Hence, the IV index varies at regional level and contains two components. The first, $\frac{{immigrants}_{cs,2005}}{{immigrants}_{c,2005}}$, is the share of immigrants from country *c* living in region *s* in 2005, and should explain, at least in part, the current distribution of immigrants across Italy’s regions. The other component, ${immigrants}_{c2005-s}$, denotes the total number of immigrants coming from country *c* to Italy in 2005 (minus the contribution of region *s* to this total); therefore, it is assumed that the nationalities with the highest number of immigrants in 2005 contributed the most in determining the current presence of immigrants.

The identifying assumption for the validity of the instrumental variable is that local factors that attracted migrants from different nationalities in the past are not correlated with current public opinion on migration, controlling for the correlates included in the final specification of the model. Some factors that could explain persistent regional differences are the levels of unemployment, gross domestic products, or levels of crime, which are included among the regressors in the final specification of the model.

A likely drawback of using this instrument is that economic conditions, which likely influence both the share of immigrants and public opinion towards immigrants, often occur at the NUTS3/province level, whereas the ESS data only pertain to the NUTS2/region level.

Unfortunately, in the previous and subsequent waves of the survey (i.e., ESS2014, ESS2018), the data are aggregated at the NUTS1 level for Italy, thus preventing a dynamic analysis of public opinion towards immigrants in relation to changes in the regional economic conditions.

Since the number of immigrants used in most previous studies may not adequately represent the “ethnic threat” of competition in the labor market, this work considered the impact of the percentage of unemployed immigrants on public opinion. In this case, reverse causality should represent a lower concern, although one could argue that ethnic penalties in accessing employment and earnings are higher when, due to racial prejudices, employers prefer native workers to immigrants. The data on unemployed (or low-income) immigrants are drawn from the Labour Force Survey of 2016. Alternative measures of immigration (i.e., the numbers of foreigners migrating to Italy and their countries of origin) are provided by ISTAT datasets.

The design weights provided in the ESS data that correct for differences in sampling were applied.

### Variables and Data

The data in Table 1 are the mean scores reported for each of the survey questions across the various European countries. As reported in previous evidence (Esipova et al., 2015) Italy is one of the European countries that is more averse to immigrants; only the Russian Federation, the Czech Republic, and Hungary report a lower willingness to accept immigrants.

**Table 1:** Anti-immigrant sentiments in the ESS countries

Country	Economic threat (question i^a)^) mean (std. err.)	Cultural threat (question ii^b)^) mean (std. err.)	Overall threat (question iii^c)^) mean (std. err.)
Austria	4.659 (0.061)	4.533(0.063)	4.203(0.056)
Belgium	4.971(0.055)	5.932(0.055)	5.107(0.049)
Switzerland	6.064(0.056)	6.107(0.060)	5.500(0.051)
Czech Republic	**3.907(0.057)**	**3.492(0.055)**	3.665(0.053)
Germany	5.806 (0.047)	5.944(0.050)	5.174(0.045)
Estonia	4.508(0.055)	4.918(0.057)	4.251(0.050)
Spain	5.455(0.059)	6.374(0.057)	5.517(0.055)
Finland	5.467(0.053)	6.964(0.048)	5.574(0.049)
France	4.820(0.061)	5.231(0.068)	4.802(0.056)
Great Britain	5.673(0.062)	5.661(0.068)	5.421(0.066)
Hungary	**3.089(0.061)**	**3.632(0.065)**	**3.527(0.057)**
Ireland	5.770(0.053)	6.029(0.052)	6.028(0.052)
Israel	4.981(0.066)	5.228(0.065)	4.825(0.064)
Italy	4.162(0.056)	4.349(0.057)	3.535(0.051)
Iceland	6.745(0.070)	7.286(0.069)	7.085(0.066)
Lithuania	5.036(0.065)	4.675(0.068)	4.649(0.062)
Norway	5.635(0.055)	5.923(0.062)	5.635(0.052)
Poland	5.107(0.067)	5.614(0.063)	5.465 (0.052)
Portugal	5.712(0.100)	6.139 (0.098)	5.300(0.088)
Russian Federation	**3.663(0.055)**	**3.507(0.054)**	**3.379(0.051)**
Sweden	5.754 (0.059)	6.946(0.059)	6.270(0.058)
Slovenia	**3.958(0.073)**	4.728(0.074)	4.377(0.066)

Respondents were asked whether they shared each sentence on a scale 0–10; lower scores reveal higher perceptions of ethnic threat and are associated here with higher anti-immigrant sentiments. Values below the scores reported for Italy are in bold

^a)^Question i: “immigration is good or bad for the country’s economy”

^b)^Question ii: “country’s cultural life is undermined or enriched by immigrants”

^c)^Question iii: “immigrants make the country a worse or better place to live”

Values below the scores reported for Italy are in bold

Considering only the ESS sample drawn from Italy, Table 2 provides summary statistics and a complete description of the variables used in the work.

**Table 2:** Summary statistics—ESS data (Italy, 2235 observations)

Variables	Variable description	Mean (std err)
Dependent variable: anti-immigrant sentiments	Respondents indicated on a scale 0–10 whether they shared the following sentences: “Immigration is good or bad for the country’s economy,” “The country’s cultural life is undermined or enriched by immigrants,” “Immigrants make the country a worse or better place to live”. The mean score varies from 0 to 10 (lower scores reveal higher perceptions of ethnic threat)	4.05 (0.051)
Age	Continuous variable (years)	50.447 (0.372)
Trust	Respondents were asked to indicate on a scale 0–10 whether they shared the following sentences: “most people can be trusted or you can’t be too careful”; “most people try to take advantage of you, or try to be fair”; “most of the time people are helpful or they are mostly looking out for themselves.” The mean score varies from 0 (low trust) to 10 (high trust)	4.515 (0.041)
Religion	Respondents answered the following questions on a scale 0–10: “How often do you attend religious services apart from special occasions,” “How often do you pray apart from at religious services.” The mean score varies from 0 (every day) to 10 (never)	4.369 (0.042)
Prejudices	Respondents were asked to indicate whether they shared the following sentences: “Gays and lesbians are free to live life as they wish,” “I am ashamed if a close family member is gay or lesbian.” The mean score reported varies from 1 to 5 (higher values indicate higher prejudices)	2.225 (0.021)
Domicile	Domicile, respondent’s description: 1 (big city), …5 (farm or home in countryside)	3.223 (0.021)
Economic conditions	Feeling about household’s income nowadays on a scale from 1 (living comfortably) to 4 (very difficult)	2.145 (0.018)
Immigrants (IMM)	Regional index calculated using microdata from the Labour Force Survey carried out in 2016: it measures the percentage of population born abroad (at regional level)	4.628 (0.519)
Unemployed immigrants (UNIMM)	Regional index calculated using microdata from the Labour Force Survey carried out in 2016: it indicates the percentage of unemployed among people born abroad (at regional level)	9.537 (0.738)
Unemployment rate	Eurostat 2016	12.195 (1.258)
IV	Instrumental variable described in Sect. 3.1	6.473 (0.104)
Crime rate	Percentages of crimes committed by foreigners at regional level compared to the number of resident foreigners	5.34 (0.02)
**Ordinal variables**	**Variable description**	**%**
Female	Dummy equal to 1 if female, 0 if male	51.44
Foreign born	Dummy equal to 1 if foreign born, 0 otherwise	8.08
Ethnic	Dummy equal to 1 if belonging to an ethnic minority, 0 otherwise	3.01
Unemployed	Dummy equal to 1 if unemployed, 0 otherwise	8.29
Discriminated	Dummy equal to 1 if the respondent is a member of a group discriminated against, 0 otherwise	8.11
Education Primary school or less Secondary school Tertiary education	Education levels according to Isced Classification	42.91 43.9 12.55
Occupation^a)^ Manager/professionals Clerks Elementary occupation Armed forces No occupation	Occupation according to ISCO 08 code	48.58 22.88 6.32 0.39 21.84

^a^^)^“Manager/professionals” includes the following: managers, senior officials and legislators, technicians; “Clerks” includes the following: clerks, service and sales workers, skilled agricultural, fishery and forestry workers, craft and related trade workers; “Elementary occupation” includes the following: plant and machine operators and assemblers, elementary occupation

The variable “anti-immigrant sentiments” was obtained by averaging the scores reported from the three survey questions above. The scores are highly correlated (Cronbach’s reliability coefficient alpha was measured as 0.89) and the mean for economic and cultural items is nearly the same (Table 1). This suggests that nothing is lost by using the total index which covers all aspects of immigrant evaluation as the dependent variable in the analysis that follows.

The set of individual-level variables includes age as a continuous variable, female coded as 1 for females, 0 for males.

Markaki and Longhi (2013) report that women are more likely to view immigrants as an economic threat while men are more likely to consider immigrants as a cultural threat. Such result seems to be in line with competition theory arguments; more specifically, we should expect that women would feel more economically threatened from immigration than men for their weaker position in the labor market (Rustembach, 2007; Hainmueller and Hiscox, 2007). However, no gender differences are found in many other studies (Dustmann & Preston, 2007; Fetzer, 2000; Sobczak, 2007).

Age may influence individual opinions for several reasons. Firstly, age marks the position of individuals in their economic lyfe (Dustman and Preston, 2007). From an economic perspective, theoretical research suggests that old individuals should be more open to immigration than younger ones (Rustenbach, 2007). In fact, assuming that immigration is mainly labor migration, immigrants could be considered to be substitutes to workers and complements to (older) capital owners (Benhabib, 1996).

Age also may capture cohort effects (Dustman and Preston, 2007). “*If racial stereotypes are the product of preadult socialization, then the historical era in which respondents come of age is likely to have a strong impact on their attitudes on a multitude of subjects*” (Burns & Gimpel, 2000, p.12). Therefore, generational differences in racial attitudes may exist.

Regarding age, some works report that young people are more hostile towards immigrants (Card et al., 2005; Sobczak, 2007), whereas other studies find a positive relationship between age and anti-immigrant sentiment (Burns & Gimpel, 2000) arguing that older individuals are more likely to support exclusion of out-groups (Gorodzeisky,^10^ 2011).

The variables “ethnic,” “foreign born,” and “discriminated” were added to determine whether the respondents either belong to discriminated groups or suffer discrimination.

Following the main research hypotheses, the set of individual predictors also includes education level (primary school or less, secondary school, and tertiary education); a dummy coded as 1 for unemployed status and 0 otherwise; individual occupation if employed coded considering the ISCO classification (elementary occupations, armed forces, clerks, professionals, and managers); and a variable denoting whether a respondent lives comfortably on his or her family income.

Further regressors were included in order to represent *personal value preferences* and *beliefs*. These variables which traditionally belong to social-psychological ways of studying prejudice are mainly added as control variables as their exclusion could affect the outcome of interest. More specifically, an indicator of individual prejudices was obtained by adding scores for the statements “Gay men and lesbians should be free to live their own life as they wish” and “If a close family member were a gay man or a lesbian, I would feel ashamed.”

In the ESS8 data, interpersonal trust is elicited from the statements “Most people can be trusted, or you can’t be too careful in dealing with people,” “Most people would try to take advantage of you if they got the chance, or they would try to be fair,”, and “People try to be helpful most of the time, or they are mostly looking out for themselves.” Respondents measure each claim on a scale of 0 to 10. The mean score reported on the three items is included as a correlate with higher values representing higher levels of interpersonal trust (the Cronbach’s reliability coefficient alpha for the summative scale was 0.81).

Numerous scholars in different fields have found that religion induces negative attitudes towards immigrants, while others have found that it fuels feelings of compassion (Bloom et al., 2015). Religious diversity may represent an important issue given that Italy is considered the center of Catholicism. Hence, the scores reported on the survey questions “How religious would you say you are?,” “How often do you attend religious services apart from special occasions?,” and “How often do you pray apart from when you are at religious services?” are summed and included as one variable representing religious sentiment (the Cronbach’s reliability coefficient alpha for the scale was 0.83).

Let us consider the set of regional variables. The percentage of immigrants compared to natives in each region and the percentage of unemployed people among immigrants represent the crucial explanatory variables for the purposes of this study. These indicators were obtained from microdata drawn from the 2016 Labour Force Survey for Italy.^11^

Other regional predictors are the unemployment rate, the gross domestic product, and the percentage of crimes committed by foreigners—compared to the number of immigrants—as reported to judicial authorities by the police force (Istat, 2016). The last measure captures views that are quite widespread in Italy (Barone et al., 2016) that immigration increases the crime rate.^12^ This index also captures the problem of irregular immigration,^13^ which is considered a likely source of measurement error in assessing the impact of migration flows on public opinion.

Table 3 analyzes the correlation between the variables in Table 2; each coefficient measures the degree of association between two variables. The results show that anti-immigrant sentiments are significantly correlated with all individual predictors (except gender) and with contextual factors. Being born abroad or belonging to an ethnic minority is positively associated with unemployment and low income conditions. A significant correlation also emerges between GDP per capita, unemployment, and crime rate at a regional level.

**Table 3:** Correlation matrix

Vars	1	2	3	4	5	6	7	8	9	10	11	12	13	14	15	16	17	18	19	20	21	22
1	**1**																					
2	** − 0.12**	**1**																				
3	0.01	0.03	**1**																			
4	**0.24**	** − 0.14**	0.03	**1**																		
5	**0.15**	** − 0.08**	− 0.02	**0.40**	**1**																	
6	** − 0.22**	**0.38**	0.02	0.03	0.03*	**1**																
7	**0.38**	** − 0.06**	− 0.01	0.01	** − 0.02**	** − 0.14**	**1**															
8	**0.06**	** − 0.29**	** − 0.21**	− 0.02	− 0.01	** − 0.18**	0.01	**1**														
9	** − 0.19**	**0.27**	** − 0.05**	**0.09**	**0.08**	**0.27**	** − 0.05**	** − 0.2**	**1**													
10	** − 0.06**	− 0.03	− 0.01	− 0.02	− 0.02	**0.05**	0.02	0.01	− 0.004	**1**												
11	**0.05**	** − 0.06**	− 0.01	**0.26**	**0.27**	**0.02**	− 0.04	− 0.04*	0.08*	** − 0.02**	**1**											
12	** − 0.07**	** − **0.19	− 0.03	**0.08**	0.003	0.01	− 0.07	**0.02**	− 0.03	0.03	− 0.05	**1**										
13	**0.09**	− 0.03	**0.14**	** − 0.09**	** − 0.06**	** − 0.22**	0.05**	− 0.04*	** − 0.11**	− 0.05*	0.03	− 0.02	**1**									
14	− **0.06**	**0.08**	** − 0.11**	0.02	0.03	**0.12**	− 0.03	0.01	0.04**	0.01	− 0.004	− 0.01	** − 0.25**	**1**								
15	− 0.01	− 0.01	− **0.05**	− 0.02	0.03	− 0.03	0.001	0.02	0.02	− 0.01	− 0.02	− 0.02	**0.06**	− 0.02	**1**							
16	− **0.21**	0.03	**0.05**	**0.12**	**0.07**	**0.29**	** − 0.19**	** − 0.07**	**0.13**	0.01	** − 0.09**	**0.25**	** − 0.17**	0.04*	− 0.04**	**1**						
17	** − 0.07**	− 0.02	− 0.003	0.02	0.02	**0.06**	0.01	− 0.07	− 0.02	**0.09**	0.01	0.02	− 0.02	0.02	− 0.002	0.006	**1**					
18	** − 0.16**	** − 0.06**	− 0.04*	** − 0.07**	− 0.05**	**0.09**	** − 0.08**	** − 0.11**	− 0.01	0.03	**0.05**	**0.20**	**0.06**	** − 0.04**	0.02	**0.25**	**0.23**	**1**				
19	** − 0.13**	− 0.04*	− 0.04*	− 0.03*	− **0.05**	**0.1**	− 0.03	0.02	− 0.03	0.05**	0.03	**0.13**	0.03	− 0.03	0.01	**0.16**	**0.04**	**0.45**	**1**			
20	**0.18**	**0.06**	**0.05**	**0.07**	0.04*	** − 0.10**	**0.11**	**0.11**	− 0.01	** − 0.08**	− 0.03	** − 0.17**	− 0.02	0.01	− 0.04*	** − 0.24**	** − 0.32**	** − 0.81**	** − 0.50**	**1**		
21	**0.15**	**0.05**	0.02	**0.06**	**0.04**	** − 0.093**	**0.094**	**0.11**	− 0.002	** − 0.05**	** − **0.02	** − 0.17**	− 0.03	0.02	− 0.03	− **0.23**	** − 0.20**	** − 0.61**	** − 0.47**	**0.85**	**1**	
22	**0.14**	**0.05**	0.03	0.04*	**0**.004	** − 0.08**	**0.06**	**0.11**	** − 0.02**	− 0.02	0.02	** − 0.14**	− 0.02	0.04*	− 0.04*	− **0.19**	** − 0.21**	** − 0.69**	** − 0.38**	**0.74**	**0.89**	**1**

Legend: 1 = anti-immigrant sentiments; 2 = age; 3 = female; 4 = foreign born; 5 = ethnic; 6 = primary school; 7 = trust; 8 = religion; 9 = prejudice; 10 = domicile; 11 = discriminated; 12 = unemployed; 13 = manager/prof; 14 = elementary occ; 15 = armed forces; 16 = economic cond.; 17 = UNIMM; 18 = unempl. rate; 19 = crime rate; 20 = regional GDP; 21 = IMM; 22 = instrumental variable IV

Each figure reports the Spearman correlation coefficient between two variables. Figures in bold: statistically significant at 1% level. **, statistically significant at 5% level; *, statistically significant at 10% level

## Empirical Models, Results, and Discussion

### Empirical Models

The research hypothesis are tested by estimating the following models; each model highlights the contribution of different correlates in explaining individuals’ attitudes towards immigrants.

- Model 1: Y_i_ = β_0_ + β_1_ UNIMM_is_ + β_2_Z_i_ + u_i_
- Model 2: Y_i_ = β_0_ + β_1_ UNIMM_is_ + β_2_Z_i_ + β_3_E_i_ + u_i_
- Model 3: Y_i_ = β_0_ + β_1_ UNIMM_is_ + β_2_Z_i_ + β_3_E_i_ + β_4_Wi + u_i_
- Model 4: Y_i_ = β_0_ + β_1_ UNIMM_is_ + β_2_Z_i_ + β_3_E_i_ + β_4_Wi + β_5_P_i_ + u_i_
- Model 5: Y_i_ = β_0_ + β_1_ UNIMM_is_ + β_2_Z_i_ + β_3_E_i_ + β_4_Wi + β_5_P_i_ + β_6_ R_is_ + u_i_
- Model 6: Y_i_ = β_0_ + β_2_Z_i_ + β_3_E_i_ + β_4_ W_i_ + β_5_ P_i_ + β_6_ R_is_ + β_7_ IMM_is_ + u_i_
- Model 7: Y_i_ = β_0_ + β_2_Z_i_ + β_3_E_i_ + β_4_ W_i_ + β_5_ P_i_ + β_6_ R_is_ + γ IMM(2sls)_is_ + u_i_

Y_i_ is the variable “anti-immigrant sentiments”, u_i_ the stochastic term. The correlates in the first model include the percentage of unemployed people among immigrants in region *s* where the *i*th individual lives,^14^ UNIMM_is_ (*hypothesis* 1), respondents’ exogenous characteristics as gender, age, the squared term of age,^15^ the dummies “Foreign born,” and “Ethnic” (Z_i_). The second includes dummies (E_i_) for a person’s educational level, while the third adds controls (W_i_) for individual positioning in the labor market and economic conditions (*hypothesis 2*). Model 4 adds a vector of control variables (P_i_), i.e., personal experiences of discrimination, the size of the municipality of residence, and the dummies “Prejudice,” “Religion,” and “Trust.” Model 5 represents the “full model,” including the other regional predictors (R_is_) as the levels of unemployment, gross domestic products, and levels of crime (*hypothesis 3*).

Model 6 considers the effect of percentage of non-EU migrants (IMM) on anti-immigrant attitudes.

The empirical analysis provides ordinary least squares (OLS) estimates of models 1–6, then investigates endogeneity issues regarding the migration variable “IMM”; Model 7, in fact, concerns 2 stage least squares estimates (2SLS).^16^

**Table 4:** Perceptions of ethnic threat in Italy^a)^—regression on ESS data (2325 observations)

Variables	Coeff. (std.err) MODEL 1	Coeff. (std. err.) MODEL 2	Coeff. (std.err) MODEL 3	Coeff. (std.err) MODEL 4	Coeff. (std.err) MODEL 5	Coeff. (std.err.) MODEL 6	Coeff. (std.err.) MODEL 7 2LS estimates	First-stage regression Dep. Var.: IMM
UNIMM	− 0.066***(0.015)	− 0.057***(0.014)	− 0.056***(0.014)	− 0.059***(0.013)	− 0.048***(0.015)			
Age	− 0.017 (0.014)	− 0.030(0.014)**	− 0.025*(0.014)	− 0.027*(0.013)	− 0.029**(0.013)	− 0.029**(0.013)	− 0.028**(0.013)	0.004 (0.006)
Age squared	0.00003(0.0001)	0.0003**(0.0001)	0.0002 (0.0001)	0.0003**(0.0001)	0.0003**(0.0001)	0.0003**(0.0001)	0.0003**(0.0001)	− 0.00005(0.00006)
Female	0.056(0.097)	0.054(0.095)	0.034(0.097)	− 0.034(0.092)	− 0.058(0.092)	− 0.066(0.093)	− 0.038(0.096)	− 0.035(0.041)
Foreign born	1.709***(0.194)	1.879***(0.193)	2.088***(0.196)	2.152***(0.191)	2.118***(0.191)	2.091***(0.191)	2.102***(0.197)	0.011(0.077)
Ethnic	1.096***(0.295)	1.205***(0.297)	1.251***(0.297)	1.492***(0.291)	1.477***(0.292)	1.468***(0.294)	1.397***(0.305)	0.231*(0.130)
Primary school		− 0.980***(0.111)	− 0.661***(0.119)	− 0.459***(0.112)	− 0.419***(0.112)	− 0.439***(0.113)	− 0.448***(0.114)	0.05(0.048)
Tertiary education		0.638***(0.152)	0.349**(0.157)	0.108(0.146)	0.111(0.146)	0.088(0.146)	0.117(0.149)	− 0.05(0.065)
Unemployed			− 0.416**(0.179)	− 0.330**(0.168)	− 0.286*(0.168)	− 0.283*(0.168)	− 0.254(0.172)	− 0.066(0.069)
Manager/prof			0.211*(0.130)	0.206*(0.121)	0.225*(0.121)	0.230**(0.121)	0.229*(0.122)	− 0.072(0.055)
Elementary occup			− 0.217(0.200)	− 0.177(0.179)	− 0.211(0.178)	− 0.231(0.179)	− 0.169(0.184)	0.050(0.049)
Armed forces			− 0.858(0.932)	− 0.677(0.891)	− 0.668(0.927)	− 0.619(0.906)	− 0.628(0.927)	0.117(0.274)
Economic conditions			− 0.514***(0.064)	− 0.308***(0.061)	− 0.265***(0.062)	− 0.244***(0.062)	− 0.247***(0.063)	− 0.019(0.027)
Trust				0.393***(0.025)	0.395***(0.025)	0.395***(0.025)	0.392***(0.025)	0.021**(0.010)
Religion				− 0.009(0.026)	− 0.015(0.026)	− 0.010(0.026)	− 0.012(0.026)	0.003(0.011)
Prejudice				− 0.479***(0.052)	− 0.481***(0.052)	− 0.469***(0.052)	− 0.459***(0.052)	− 0.091(0.109)
Domicile				− 0.147***(0.044)	− 0.145***(0.044)	− 0.156***(0.044)	− 0.150***(0.044)	− 0.029(0.019)
Discriminated				0.007(0.283)	0.008(0.284)	0.013(0.283)	0.007(0.287)	− 0.091(0.109)
Unempl. rate					0.006(0.019)	0.005(0.019)	− 0.0002 (0.019)	− 0.027***(0.008)
Crime rate					− 0.14***(0.04)	− 0.157**(0.039)	− 0.027(0.081)	− 0.191***(0.021)
Regional GDP					0.015(0.015)	0.044**(0.021)	− 0.096(0.080)	0.249***(0.07)
IMM						− 0.063(0.050)		
IMM2sls							0.379(0.242)	
IV								0.07***(0.005)
F-test	34.52	39.62	29.30	35.66	34.17	33.81		156.095^b)^
n. parameters	7	10	18	33	36	36	36	
adj. *R*-squared*100	8.08	13.07	16.81	29.20	30.86	30.58	27.79	
Wald test							1179.42	

^a)^A higher value of the dependent variable indicates a lower perception of the ethnic threat and is associated here with a greater acceptance of immigrants

^b)^F-test for the significance of the instrument excluded from the structural equation

^*^Statistical significant at 10% level

^**^Statistical significant at 5% level

^***^Statistical significant at 1% level. Notes: Robust standard errors. The design weights provided in the ESS data which correct for differences in sampling are applied

### Results and Discussion

This section reports the estimation results. Firstly, I examine what factors shape residents’ attitudes towards immigrants, with particular reference to the argumentations of competition theory (models 1–5). Second, I focus on two factors which, in my opinion, need further attention: the impact of the mere size of immigrants on public fears of “ethnic threat” and endogeneity issues in evaluating the impact of the migration flows.

The results of the estimation of models 1–5 are reported in Table 4, columns I–V.

The first finding was the importance of the contextual variables, e.g., the percentage of unemployed immigrants and local economic conditions. Overall, hostility towards immigrants significantly increases when the share of unemployed immigrants increases, as predicted by competition theory (hypothesis 1); the relative coefficient is quite stable across the different models (it is about 6%), slightly decreases when regional controls are included in column 5, but remains statistically significant at the 1% level.

Let us consider the other regional correlates in model 5. Attitudes towards immigrants across European countries have often been found weakly related to local unemployment or income (Card et al., 2005; Esipova et al., 2015). On the contrary, in this work, GDP per capita and the regional unemployment rate significantly affect public opinion (when included separately in the model). In fact, in preliminary estimates—available on request—the estimated coefficient on GDP was 0.017 (t-stat = 2.25) whereas the coefficient on the unemployment rate was − 0.021 (t-stat = 2.40). In model 5, however, the coefficients on the unemployment rate and gross domestic product are not significant because of collinearity problems. Regions with high crime rate tend to be characterized by stronger anti-immigrant attitudes, thus supporting fear-based arguments against crime.

## Result 1

*Anti-immigrant sentiment is significantly and positively correlated with an increase in the percentage of unemployed immigrants in the local population and with the local unemployment rate. Evidently, anxiety regarding immigration increases when immigrants are seen as competitors for available resources and job opportunities, particularly in areas where large parts of native population are facing economic hardships. Security fears raise concerns about immigration.*

An additional result concerns the individual characteristics that explain aversion to immigrants.

Education is negatively correlated with anti-immigrant sentiment in model 2; however, this association becomes weaker controlling for working and economic conditions in model 3. The coefficient on tertiary education, in fact, decreases from 0.638 to 0.349, whereas the coefficient on primary education varies from − 0.980 to − 0.662 (“secondary school” is the excluded dummy). According to competition theory, this finding suggests that people with low education are more concerned about immigration because they compete with similarly qualified newcomers for low-skill jobs (*hypothesis 2*). Similar results have been reported in previous studies (for example, in Andreescu, 2017; Card et al., 2005; Manevska & Achterberg, 2011). Some authors argue that finding a significant association between education and hostility, also controlling for occupation (as in model 3), reveals the presence of cultural factors, i.e., not economic competition, which are positively correlated with education (e.g., open mindedness, liberal view). However, a more complete test for socio-educational elites would be to measure their responses in relation to the migration of highly qualified foreigners, given that these foreigners could be in competition with the natives.^17^

In model 3, unemployed and people who have difficulties coping on their current income are more likely to evaluate immigration as threatening whereas managers, senior officials, and professionals are more likely to view immigration as positive.

## Result 2

*Much of the variability in anti-immigrant hostility is due to individual characteristics. In line with competition theory, economic concerns about immigration increase among unemployed and low-skilled people who fall in direct competition for jobs with similarly experienced newcomers.*

An interesting broadening of this research could take into account the likely effect of the COVID-19 pandemic on attitudes towards immigrants. The pandemic, in fact, has heightened economic difficulties, especially for people with less protection in the labor market. Some authors (Clissold et al., 2020; Esses & Hamilton, 2021) have also underlined the likely impact of the pandemic on nations and individuals in terms of increasing feelings of threat and competition, uncertainty, and lack of control.

The other individual-level variables have significant and similar effects in all models, the direction of the effects being the same.

Gender does not matter while acceptance of immigrants decreases with age (at an increasing rate); the cross-sectional nature of the data, however, does not reveal whether the latter result is associated with aging or with differences across birth cohorts.

The coefficient on “domicile” suggests that anti-immigrant prejudice decreases in large urban areas; to this regard, literature is scarce and not conclusive (Lahdelma, 2020). Markaki and Longhi find a similar result and argue that, if more immigrants arrive in big cities searching for a job and if intergroup contact increases because of higher population density, this result is consistent with contact theory. In line with competition theory, however, one could argue that arrivals of immigrants and refugees are more visible in villages than in cities, and the increasing competition for local public services and in the labor market increases residents’ hostility.

Overall, ethnic minorities and non-natives are less likely to express anti-immigrant sentiments, as hypothesized by Fetzer (2000) and confirmed by Rustenbach (2010). It is quite intuitive that immigrants, or children of immigrants, are more likely to accept other cultures since they have themselves been exposed to different cultures (Goldstein & Peters, 2014); they should also be more likely to understand the needs and difficulties of immigrants and to become familiar with them.

The attitudinal predictors described in Sect. 3.2 are added in model 4. The results indicate a negative and strong association between feelings towards immigrants and prejudices regarding LGBT, as the relative coefficient is equal to − 0.479 (t-stat = 9.211). This seems to reveal a more general attitude to discriminate against out-group members. Similarly, anti-immigrant attitudes are consistently expressed by people with low levels of interpersonal trust; Rustenbach (2010) reports a similar finding and argues that people who trust others do not follow racial or cultural stereotypes when forming their opinions about other people’s trustworthiness.

Direct personal experience of discrimination does not influence a person’s sentiments towards immigrants, contrary to predictors in line with cultural marginal theory (Fetzer, 2000; Rustenbach, 2010). The available data, however, are not suitable for the kinds of the further tests generally carried out to support cultural marginal or contact theories. In fact, ESS8 data do not contain information on any contacts—casual or personal—between respondents and immigrants, or on whether respondents had friends, parents, or co-workers of a different race or ethnic origin than their own.

A crucial variable for this analysis is the share of immigrants as a likely “ethnic threat.” The estimates in columns 6–7 address two methodological issues since (i) display the results obtained including among the explanatory variables the immigration size rather than the size of unemployed immigrants and (ii) enable any endogeneity issues in the migration flows to be investigated.

In model 6, the coefficient estimated on the percentage of non-EU immigrants is not statistically significant.Preliminary estimates have also considered the overall number of immigrants—irrespectively of their ethnic origins—as well as the percentage of immigrants from Asia and Africa; such estimates have reported similar results.^18^ Overall, this finding is consistent with most previous studies (Card et al, 2005; Jetten et al., 2015; Moscatelli et al., 2014; Papademetriou & Banulescu-Bogdan, 2016). As argued above, not only the size of immigration is probably a weak indicator of competition among immigrants and natives, but it might also have the opposite effect on anti-immigrant sentiment according to contact theory. Although this work does not account for any interpersonal contacts between respondents and immigrants, one could argue that meaningful personal contacts—and not casual contacts—are more likely to occur in areas with larger immigrant populations (Andreescu, 2017).

## Result 3

*Natives’ concerns about immigration do not automatically increase with the increase of immigrants. Rather, it is the percentage of unemployed immigrants that increases feelings of threat from immigration (see Result 1).*

Hence, researchers in this context should take into account migration’s composition and its impact in the local context.

In Sect. 3, I argued that the finding of a weak and insignificant effect of the immigration size on residents’ opinion could be due to endogeneity in the number of immigrants. For example, unobserved local productivity shocks may attract more immigrants and, at the same time, lower competition in the labor market, which—according to competition theory—would entail lower perceptions of ethnic threat in the local population. At the same time, immigrants might choose to live where they feel more accepted. Measurement errors may also occur. To control for local economic conditions, the set of regressors in the final specification of the model includes regional unemployment rates and gross domestic products. The method of instrumental variables (IV) and 2stage least squares (2SLS) described in Sect. 3.1 provide a further solution to potential endogeneity problems.

Hence, Table 4 reports 2SLS estimates (model 7) considering the variable IMM as endogenous and instrumented by the IV variable described in Sect. 3.1, i.e., the distribution among regions of immigrants according to their origins in the past. The last column reports the first-stage regression of the potentially endogenous variable IMM on all the other regressors included in model 7 and on the IV variable (the relative coefficient is 0.07, the t-stat is 12.53). Overall, the evidence suggests that the IV variable is not a weak instrument since the F-stat for the significance of the instrument excluded from the structural equation is 156.95, i.e., well above the rule of thumb value of 10.

Let us now compare OLS estimates (model 6) and 2SLS estimates (model 7). In both cases, the percentage of non-EU immigrants is not statistically significant. A Durbin-Wu-Hausman test suggests that the null hypothesis that the immigrant flow is exogenous should not be rejected (the F-stat is 3.57, and the *p*-value is 0.059). Hence, OLS estimates (model 6) represent the final empirical specification of the model.

When considering the impact of the percentage of unemployed immigrants on public opinion, endogeneity of the UNIMM variable should be of less concern, as specified in Sect. 3.1. However, further estimates are provided to test for endogeneity; 2SLS estimates and the first-stage regression are reported in the Appendix Table 6. Once again, the evidence suggests that the chosen IV is not a weak instrument (the F-stat is equal to 14.04) and a Durbin-Wu-Hausman test indicates that the null hypothesis that the percentage of unemployed immigrants is exogenous should not be rejected at 5% level (F-stat = 1.98). Hence, OLS estimates (model 5) represent the final specification of the model.

## Result 4

*According to the estimates in this work, endogeneity issues of migration flows do not pose serious concerns in evaluating the impact on attitudes towards immigrants.*

As noted in Sect. 3.1, a possible weakness of the instrument used in this work is that economic conditions, which likely influence both the share of immigrants and residents’ attitudes towards immigrants, often occur at the province level, whereas the ESS data only pertain to the region level.

This work has mainly focused on residents’ opinion about immigration since residents (whether born in the host country or not) share the effects of immigration. However, it is reasonable to assume—and confirmed in Table 4—that foreign-born individuals and minorities may have different opinions from “Italian natives,” which might affect our results.

Therefore, Table 5 reports further estimates of the empirical models in Sect. 4.1, excluding ethnic minorities and non-natives from the sample. Overall, the main results are substantially the same^19^; however, the coefficient on the share of immigrants is lower. For example, considering the most parsimonious version of the model (first column), the coefficient on the share of unemployed immigrants varies from − 0.066, in Table 4, to − 0.056 in Table 5. In a parallel analysis, carried out only on a subsample of foreign-born individuals,^20^ the estimated coefficient on the share of unemployed immigrants was − 0.19. This suggests that ethnic minorities and non-natives feel more threatened than Italian natives by newcomers. This result is not surprising given that Table 3 would seem to highlight that being born abroad or belonging to an ethnic minority is positively associated with unemployment and low income conditions.

**Table 5:** Perceptions of Ethnic threat in Italy^a)^—regression on ESS data (2118 observations-ethnic minorities and non-natives are not in the sample)

Variables	Coeff. (std.err) I	Coeff. (std.err) II	Coeff. (std.err) III	Coeff. (std.err) IV	Coeff. (std.err.) V
UNIMM	− 0.056***(0.015)	− 0.047***(0.015)	− 0.048***(0.015)	− 0.039***(0.015)	
Age	− 0.018(0.015)	− 0.025*(0.015)	− 0.026*(0.014)	− 0.027*(0.013)	− 0.027**(0.013)
Age squared	− 0.00005(0.0001)	0.0002(0.0001)	0.0003**(0.0001)	0.0003**(0.0001)	0.0003**(0.0001)
Female	0.035(0.102)	0.021(0.102)	0.003(0.003)	− 0.019(0.096)	− 0.022(0.096)
Primary school		− 0.704***(0.129)	− 0.498***(0.120)	− 0.453***(0.120)	− 0.470***(0.121)
Tertiary education		0.355**(0.166)	0.117(0.153)	0.110(0.153)	0.092(0.154)
Unemployed		− 0.430**(0.196)	− 0.301*(0.184)	− 0.243 (0.153)	− 0.234(0.183)
Manager/prof		0.203(0.135)	0.204*(0.124)	0.228*(0.124)	0.232*(0.124)
Elementary occup		− 0.223(0.209)	− 0.190(0.186)	− 0.223(0.183)	− 0.241(0.185)
Armed forces		− 0.946(1.043)	− 0.722(1.023)	− 0.747(1.068)	− 0.666(1.053)
Economic cond		− 0.546***(0.068)	− 0.322***(0.064)	− 0.284***(0.065)	− 0.267***(0.065)
Trust			0.407***(0.025)	0.409***(0.025)	0.409***(0.025)
Religion			0.014(0.027)	0.011(0.027)	0.015(0.028)
Prejudice			0.469(0.381)	− 0.514***(0.054)	− 0.506***(0.054)
Domicile			− 0.139***(0.046)	− 0.134***(0.046)	− 0.143***(0.046)
Discriminated			0.469(0.381)	0.463(0.379)	0.473(0.376)
Unempl. rate				0.006(0.019)	0.005(0.019)
Crime rate				− 0.163***(0.041)	− 0.167***(0.043)
Regional GDP				0.012(0.016)	0.029(0.022)
IMM					− 0.031(0.048)
F-test	15.04	18.80	29.86	28.72	28.26
n. parameters	4	16	30	33	33
adj. *R*-squared*100	2.57	11.17	26.17	26.84	27.75

^a)^A higher value of the dependent variable indicates a lower perception of the ethnic threat and is associated here with a greater acceptance of immigrants

^*^Statistical significant at 10% level

^**^Statistical significant at 5% level

^***^Statistical significant at 1% level. Notes: Robust standard errors. The design weights provided in the ESS data which correct for differences in sampling are applied

## Result 5

*In line with competition theory, one could argue that ethnic minorities and earlier immigrants are in a more vulnerable position in the labor market and see themselves in a stronger competition with new immigrants.*

Overall, whether or not one has been a migrant oneself seems to be an important feature in explaining attitudes towards immigrants. On the one hand, having a migration background influences the formation of positive opinions about immigration; on the other hand, the influx of new immigrants, by increasing the competition for jobs and resources, could lead to tensions between new and old immigrants who often work for low wages. Therefore, in analyzing immigrants’ opinions about immigration, there may be unresolved questions which future research could take into account, particularly in contexts where the percentage of people with a migratory background is quite high (Becker, 2019).

## Concluding Remarks

Immigration is an important issue in the public policy debate in Italy as well as in Europe, and people’s fears of immigration are often taken into account during electoral campaigns. After a short theoretical discussion about the reasons that may influence people’s perception of an “ethnic threat,” this work has investigated anti-immigrant sentiment in Italy.

The empirical analysis has focused on data drawn from the European Social Survey-Eight Sweep, conducted in 2016. Individuals were asked their views on whether immigration is beneficial for the economy, for cultural life, and whether immigration improves the quality of life in the receiving country. According to the ESS data, Italy is one of the European countries more concerned about immigration.

In this work, the total index covering all aspects of how immigrants were evaluated in the survey was related to the individual and contextual factors expected to influence public perceptions of immigrants.

This research focused mainly on the effects of the presence of immigrants on residents’ perceptions of an “ethnic threat.” Since immigrants tend to migrate to areas where communities of immigrants from the same country of origin have already settled, the potential endogeneity of the share of foreigners in the region population has been addressed using immigrant enclaves.

Consistently with the economic perspective, the results indicate that it is the percentage of unemployed immigrants—and not the mere size of immigrants—that increases feelings of threat from immigration. Endogeneity issues do not affect these results.

Overall, findings support the economic self-interest perspective, rooted in ethnic competition theory, in that people are more likely to express negative views about immigrants when they feel threatened in their economic position. Indeed, people with a low level of education, poor economic conditions, and living in regions with high unemployment rates are less favorable to immigration flows.

Finally, the results show that attitudes towards immigration vary systematically according to socio-demographic variables—i.e., age, size of the municipality of residence, place of birth, and ethnicity—and attitudinal predictors. Distrust and fear of increasing criminality also emerge as important factors associated with anti-immigrant sentiments.

Therefore, policy makers need to make considerably more effort to be in touch with the concerns of the general public.

It has been said that Italy has not adopted active policies of labor recruitment, while the managing of the migration flows has been mainly characterized by periodic regularizations of the illegal workers. However, this does not mean that the role of foreign workers is marginal or insignificant in the Italian context. To this regard, the Italian government should raise awareness that immigration can be a means of increasing economic growth and competitiveness. The criteria for the admission of immigrants should be shown to take into account the current state of the job market; this might lower people’s fears in the labor market thus favoring real integration into society.

Furthermore, the resident population should also be reassured that immigration management, including security questions, is under the control of the government.

The empirical analysis has some limitations that leave scope for future research.

First, the European Social Survey provides cross-sectional data in which an independent sample is collected in each wave, and this does not permit a dynamic analysis of attitudes towards immigrants due to changes in economic conditions. Moreover, in the previous and subsequent waves of the survey (i.e., ESS2014, ESS2018), the data are aggregated at the NUTS1 level for Italy, thus preventing a further analysis at a regional level.

Additional insights, in other EU countries besides Italy, could be drawn from further and more recent data in the COVID-19 pandemic period. Some reports suggest that ethnic minority groups have been more affected by SARS-CoV-2 infection (for example, Rimmer, 2020; Killerby et al., 2020; Price-Haywood et al., 2020; Fabiani et al., 2021). At the same time, the pandemic might also have affected residents’ attitudes towards immigrants. In this regard, in fact, some authors (Clissold et al., 2020; Esses & Hamilton, 2021) have underlined the likely impact of the pandemic on nations and individuals in terms of increasing feelings of threat and competition, uncertainty, and lack of control.

Second, the questions that form the composite measure of anti-immigrant sentiments in the work refer to immigrants in general, without differentiating between legal, illegal, and refugees. Hence, it would be interesting to differentiate between legal, illegal, and refuges and to examine whether such differences influence public opinion about immigration.

## Appendix

**Table 6:** Perceptions of ethnic threat in Italy: 2SLS estimates and first-stage regressions

Variables	Dep. variable: anti-immigrant sentiments^a)^ Coeff. (std.err)	First stage regression Dep. Var.: UNIMM Coeff. (std.err)
Age	− 0.031**(0.015)	− 0.003(0.019)
Age squared	0.0003**(0.0001)	0.00004(0.0002)
Foreign-born	2.316***(0.257)	0.512**(0.223)
Ethnic	1.613***(0.322)	0.343(0.374)
Female	− 0.033(0.105)	0.078(0.128)
Primary school	− 0.272*(0.169)	0.431**(0.160)
Tertiary education	0.241(0.188)	0.369**(0.193)
Trust	0.388***(0.027)	− 0.016(0.031)
Religion	− 0.046(0.036)	− 0.083**(0.035)
Prejudice	− 0.582***(0.093)	− 0.280**(0.072)
Domicile	− 0.065(0.079)	0.230***(0.060)
Discriminated	0.036(0.314)	0.149(0.326)
Unemployed	− 0.346*(0.205)	− 0.139(0.291)
Manager/prof	0.213*(0.130)	− 0.038(0.159)
Elementary occup	− 0.128(0.205)	0.252(0.255)
Armed forces	− 1.062(1.173)	− 1.049(1.232)
Economic cond	− 0.413***(0.133)	− 0.403***(0.089)
Unempl. rate	0.016(0.023)	0.065*(0.033)
Crime rate	− 0.192***(0.053)	− 0.20***(0.05)
Regional GDP	− 0.054(0.056)	− 0.127***(0.034)
UNIMM2sls	− 0.421*(0.260)	
IV		− 0.06***(0.01)
F-test^a)^		14.04
adj. *R*-squared*100	19.26	
Wald test	931.59	

Notes: Robust standard errors. The design weights provided in the ESS data which correct for differences in sampling are applied

^a)^A higher value of the dependent variable indicates a lower perception of the ethnic threat and is associated here with a greater acceptance of immigrants

^b)^F-test for the significance of the instrument excluded from the structural equation

^*^Statistical significant at 10% level

^**^Statistical significant at 5% level

^***^Statistical significant at 1% level

## Footnotes

1 Their research resulted from the discussion held during a plenary meeting of the Transatlantic Council on Migration (Rome, 2015).

2 National Institute of Statistics, ISTAT (dati.istat.it, accessed May 2022).

3 In 1998, the center-left government issued the Turco-Napolitano law in order to regularize illegal migrants and to improve their integration through regulations concernimg employment, health, and education. Overall, the center-left coalization stressed the importance of the immigrants for the domestic economy as well as the importance of a multi-ethnic culture. In 2002, the Bossi-Fini law—issued by the center-right government—regularized a large number of non-EU workers, but introduced new stringent clauses that actually limited foreigners’ possibilities to come to Italy for work. The center-right coalization was more focused on security problems (Barone et al., 2016; Colucci, 2018).

4 These indicators concern labor market mobility, health, access to nationality, family reunion, political participation, anti-discrimination, education, and permanent residence and assess whether immigrants enjoy the same rights as nationals in these dimensions. A “*Halfway favorable approach encourages the public to see immigrants as equals but as foreigners*.” https://www.mipex.eu, accessed on july, 2022.

5 Salvati et al. (2019) used data from a national survey, conducted by Istat in 2011, titled “Discrimination by gender, sexual orientation and ethnic origin.” Panichella and Ambrosini (2018) used data collected by CISF (Centro International Studi Famiglia) in 2013. Russo et al. (2019) used a longitudinal sample extracted from the Italian population from 2002 to 2008.

6 The authors report a positive and statistically significant relationship between immigrant group size and ethnic prejudice only for right-wing individuals. The present work does not investigate the impact of political orientations on opinions about immigrants since endogeneity problems could arise in the absence of adequate longitudinal data. In the Italian context, in fact, the debate between right- and left-wing political parties has been mainly characterized by opposing attitudes towards immigrations.

7 64 defined themselves as ethnic minorities.

8 See note 16 for more details on the instrumental variable approach.

9 *N* is equal to 15. Hence, the instrumental variable is constructed considering the 15 countries with the highest number of immigrants in 2005, namely Albania, China, Ecuador, Egypt, Philippines, India, Macedonia, Morocco, Peru, Poland, Romania, Senegal, Serbia, Sri Lanka, Tunisia, and Ukraine. The share of these nationalities in 2005 amounted to approximately 60% of total immigration. Few changes have occurred since 2005 (see the data reported in the introduction).

10 Gorodzeisky (2011) reports that in Israel, the perception of threat to national identity is more prevalent among older people.

11 To check robustness, preliminary estimates considered alternative indexes concerning immigration flows which were obtained from data drawn from the 2016 LFS data for Italy.

12 On this point, however, Bianchi et al. (2012) show that, controlling for endogeneity, immigration in Italy has not led to more criminality.

13 Approximately 70% of crimes committed by foreigners are committed by irregular immigrants whereas these account only for approximately 10% of total immigrants. For more details, see Bargagli and Colombo (2011).

14 Parallel analyzes were performed using both OLS with standard errors clustered at regional level and hierarchical models. Final estimates use the OLS method which produces essentially the same results.

15 The squared term is included because “age” might be non-linearly related to the outcome variable.

16 The method of instrumental variables (IV) provides a general solution to the problem of endogeneity. As discussed in Sect. 3.1, in case of endogeneity, the regressor “IMM” in “Model 6” might be correlated with the error term “u”; consequently, the OLS estimator would be inconsistent. The IV variable must satisfy two conditions: (1) it must be not correlated with current public opinion on migration—that is, the dependent variable “Yi” in model 6—after controlling for the other correlates included in the model; (2) it must be correlated with the potentially endogenous “IMM” regressor in order to provide information on it. In particular, the second condition requires the linear regression of the variable “IMM” on all the other exogenous variables of model 6 and on the variable IV, as follows: IMM_is_ =δ _0_ +δ _2_Z_i_ +δ _3_S_i_ +δ _4_ W_i_ + δ_5_ P_i_ +δ_6_R_is_ + ƟIV_is_ (first-stage regression reported in the last column of Table 4); the key assumption for the validity of the variable IV is that Ɵ ≠ 0 (usually, the coefficients on the other variables in the first stage regression are not of interest). The IV variable chosen in this work, as well as the identifying assumptions, are described in Sect. 3.1. The final 2SLS estimates are in “Model 7” where the crucial variable—representing migration flows—is the instrumented variable IMM(2SLS) obtained from the first-stage regression. OLS estimates are compared to 2SLS estimates in order to test for exogeneity (see also the Durbin-Wu-Hausman test in Sect. 4.2).

17 I thank an anonymous referee for this suggestion.

18 Estimates available on request. Symmetrically, the coefficient on the share of low-educated (or low-income) immigrants was estimated with the negative sign, confirming the main results in Table 4.

19 Similarly, a Durbin-Wu-Hausman test led to not rejecting the null hypothesis that the immigrant flow is exogenous. Estimates available on request.

20 Available upon request.
